# A Universal Approach to Prepare Reagents for DNA-Assisted Protein Analysis

**DOI:** 10.1371/journal.pone.0108061

**Published:** 2014-09-18

**Authors:** Junhong Yan, Gucci Jijuan Gu, Christian Jost, Maria Hammond, Andreas Plückthun, Ulf Landegren, Masood Kamali-Moghaddam

**Affiliations:** 1 Department of Immunology, Genetics and Pathology, Science for Life Laboratory, Uppsala University, Uppsala, Sweden; 2 Department of Genetics, Stanford University, Palo Alto, California, United States of America; 3 Department of Biochemistry, University of Zurich, Zurich, Switzerland; Deutsches Krebsforschungszentrum, Germany

## Abstract

The quality of DNA-labeled affinity probes is critical in DNA-assisted protein analyses, such as proximity ligation and extension assays, immuno-PCR, and immuno-rolling circle amplification reactions. Efficient, high-performance methods are therefore required for isolation of pure conjugates from reactions where DNA strands have been coupled to antibodies or recombinant affinity reagents. Here we describe a universal, scalable approach for preparing high-quality oligonucleotide-protein conjugates by sequentially removing any unconjugated affinity reagents and remaining free oligonucleotides from conjugation reactions. We applied the approach to generate high-quality probes using either antibodies or recombinant affinity reagents. The purified high-grade probes were used in proximity ligation assays in solution and *in* *situ*, demonstrating both augmented assay sensitivity and improved signal-to-noise ratios.

## Introduction

DNA-assisted protein analysis technologies play an increasing role in analyses of proteomes [1], and for disease diagnosis, prognosis [2] and personalized medicine [3]. For optimal performance of such analyses, high-quality and pure DNA-coupled affinity binders are required. A broad variety of methods are available for protein-DNA conjugation, in order to functionalize antibodies for DNA-assisted protein analysis. Commonly, biotinylated antibodies are coupled with oligonucleotide-modified streptavidin molecules. Alternatively, bifunctional cross-linkers can be used to covalently link antibodies to oligonucleotides with suitable chemical modifications. For instance, primary amines of antibodies can be reacted with dibenzylcyclooctyne-NHS esters to allow copper-free click chemistry to attach azide-modified oligonucleotides to the alkyne-functionalized antibodies [4]. Recently, the growing availability of new classes of affinity binders prepared by recombinant techniques, such as recombinant antibody fragments [5], affibodies [6] and designed ankyrin repeat proteins (DARPins) [7], greatly increases opportunities for DNA-assisted protein analysis. By expressing recombinant binders in fusion with SNAP protein domains, O^6^-benzylguanine (BG)-modified oligonucleotide can be covalently coupled to SNAP domains of the affinity reagents [8]. The use of suitably modified recombinant affinity reagents has the virtue that oligonucleotides can be attached to the recombinant binder at a specific site, remotely from the target binding area, and at a one-to-one (or any other desired) stoichiometry.

In the various conjugation methods, the efficiency of functionalization of affinity binders is usually maximized by employing a large excess of suitably modified oligonucleotides, thereby generating a mixture of the desired conjugates and an excess of free oligonucleotides, along with any remaining unconjugated affinity binders. In DNA-assisted protein analysis, remaining free oligonucleotides are liable to cause target-independent detection signals, increasing assay background. This is particularly deleterious in homogeneous assays where no washing steps are applied, such as in solution phase proximity ligation assays (PLA) [9], [10] or proximity extension assays (PEA) [11]. Any unconjugated binders in the detection reactions, be it antibodies or alternative scaffolds, also tend to compromise assay performance by competing with properly functionalized affinity reagents for binding to the respective targets, resulting in reduced detection signals. Present protocols for purifying conjugates may remove either free oligonucleotides or antibodies, but there is a need for a general and convenient procedure to avoid both contaminating species in one simple procedure [12].

We present in this study a general and efficient approach for purification of high-quality DNA-coupled affinity binders from free oligonucleotides and remaining unconjugated natural antibodies or alternative binding scaffolds. Alkyne-modified antibodies and azide-modified oligonucleotides were conjugated by click chemistry, and recombinant DARPin affinity reagents were coupled with BG-activated oligonucleotides via SNAP domains. The conjugation reaction mixtures were subjected to solid-phase purification, where unconjugated binders and excess oligonucleotides were removed in two sequential steps. The purified DNA-labeled affinity reagents were then used in solution phase and *in* *situ* PLA to validate the effect of the conjugation and purification protocol on sensitivity and signal-to-noise for the two assays.

## Materials and Methods

### Affinity binders, recombinant proteins and oligonucleotides

Goat anti-human IL8 (AF-208-NA) and recombinant human IL8 (208-IL-010) were purchased from RnD Systems. Anti-HER2 DARPins 9.01 and G3, in fusion with both an N-terminal RGS His-tag and a C-terminal SNAP domains, referred to as 9.01-SNAP and G3-SNAP, respectively, were expressed and prepared as described previously [13]. Oligonucleotides with suitable modifications (Table S1) were purchased from Integrated DNA Technology.

### Cell culture

The HER2-expressing human ovarian cancer cell line SK-OV-3 ((HTB-77; ATCC)) was grown in RPMI1640 medium, and the human fibroblast cell line BJhTERT was grown in Minimum Essential Medium (MEM). Both media were supplemented with 10% fetal bovine serum (FBS), 2 mM L-glutamine and 1% penicillin-streptomycin (all reagents from Sigma-Aldrich). The cells were cultured at 37°C in a humidified 5% CO_2_ environment.

### Preparation of antibody-DNA conjugates

Ten µl of antibodies (2 µg/µl reconstituted in PBS) were activated at room temperature (RT) for 30 min with a 20-fold molar excess of dibenzylcyclooctyne-NHS ester (DBCO-NHS ester, CLK-A102N, Jena Bioscience; 0.67 µl of a 4 mM solution of DBCO-NHS ester freshly dissolved in DMSO). The DBCO-activated antibodies were purified over a Zeba spin desalting column (7K MWCO, Thermo Scientific) to remove unreacted DBCO-NHS ester. After purification, the activated antibodies were mixed with a 4-fold molar excess of the azide modified oligonucleotides (Arm1_long, Arm2_long, Arm1, or Arm2, Table S1) and incubated overnight at 4°C.

### Preparation of DARPin-DNA conjugates

Thirty µl of 200 µM 5′-aminohexyl modified oligonucleotides (S3primer_long, S3block_long, S3primer and S3block, Table S1) were incubated for 2 h at 37°C with a 30-fold molar excess of an amino-reactive bifunctional cross-linker to functionalize the oligonucleotides with a benzylguanine (BG) moiety (BG-GLA-NHS (S9151S, NEB); 18 µl of a 10 mM solution BG-GLA-NHS freshly dissolved in DMSO). Excess BG-GLA-NHS was removed by separation through two 7K MWCO Zeba spin desalting columns, and the purified BG-modified oligonucleotides were collected. The DARPins 9.01-SNAP and G3-SNAP were reduced to activate the cysteine residue within the SNAP domain in 20 mM DTT for 1 h at 37°C, separately. The reduced DARPins were then mixed with a 3-fold molar excess of the purified BG-modified oligonucleotides, and incubated for 1 h at 37°C.

### Two-step purification of conjugates

To remove unconjugated protein, biotinylated capture oligonucleotides were added at a 2-fold molar excess over the partially complementary conjugation oligonucleotides (Arm1Capture for Arm1_long, Arm2Capture for Arm2_long or S3Capture for both S3primer_long and S3block_long) to the respective antibody- or DARPin-oligonucleotide conjugates, followed by incubation for 30 min at RT. The solution was then incubated for 30 min at RT with Streptavidin Sepharose High Performance (17-5113-01, GE Healthcare) using 150 nmol biotinylated capture oligonucleotides per milligram beads. After two washes with PBST (PBS containing 0.05% Tween 20) to remove unconjugated protein, 50 µl of enzymatic elution solution (1× NEB buffer 4, 1 mg/ml BSA, and 1 U/µl of the restriction enzyme MlyI (R0610S, NEB)) was added to the streptavidin Sepharose and incubated overnight at 37°C.

Next, for antibody-DNA conjugates, the supernatant from the MlyI cleavage was incubated with Dynabeads Protein G (10004D, Life Technologies) for 1 h at RT, followed by two washes with PBST to remove unconjugated oligonucleotides. The antibody-DNA conjugates were then eluted by incubating with 50 mM glycine-HCl (pH 2.5) for 5 min at RT and the eluate was immediately adjusted to pH 7 with Tris buffer (pH 8.2).

For DARPin-DNA conjugates, the supernatant from the MlyI cleavage was incubated in phosphate buffer (0.1 M phosphate, 0.5 M NaCl and 0.05% Tween 20, pH 8.3) with Dynabeads® His-Tag Isolation and Pulldown (10103D, Life Technologies) for 1 h at RT. The DARPin-DNA conjugates were washed three times in PBS to remove unconjugated oligonucleotides, and then eluted with 0.1 M imidazole in PBS for 5 min at RT.

The conjugates were quantified using the Quant-it protein assay kit (Q33210, Life Technologies), and analyzed by electrophoresis in GeneGel Excel 12.5/24 Kit (17-6000-14, GE Healthcare) at a current of 25 mA for 70 min. The gels were stained with either PlusOne DNA Silver Staining Kit (12-1150-01, GE Healthcare) or Pierce Silver Stain for Mass Spectrometry kit (24600, Thermo Scientific). After quantification and gel analysis the conjugates were supplemented with 0.1% BSA and 0.05% NaN_3_ and stored at 4°C.

### Solution phase PLA

Recombinant human IL8 was serially diluted in PLA buffer (1 mM D-biotin (Life Technologies), 0.1% BSA (Sigma-Aldrich), 0.05% Tween 20, 100 nM goat IgG (Sigma-Aldrich), 0.1 µg/µl salmon sperm DNA (Life Technologies), 5 mM EDTA in PBS), or in either 10% or 50% chicken serum prepared in PLA buffer. Individual dilution series included a negative control with no spiked antigen. Two µl of each sample were mixed with 2 µl of 60 pM PLA probes (either purified anti-IL8-Arm1_long and anti-IL8-Arm2_long conjugates, unpurified anti-IL8-Arm1 and anti-IL8-Arm2 conjugates, or a pair of purified conjugates with spiked-in anti-IL8 or Arm1 and Arm2, respectively) and incubated for 1.5 h at RT. After incubation, 1 µl of the mixture was transferred to 25 µl ligation and quantitative PCR mixture (1× PCR buffer (Quanta Biosciences), 2.5 mM MgCl_2_ (Quanta Biosciences), 0.5× Sybr Green I (Life Technologies), 0.1 µM BioFwd primer, 0.1 µM BioRev primer, 0.025 µM BioSplint, 0.08 mM ATP (Thermo Scientific), 0.2 mM dNTPs (with dUTP) (Thermo Scientific), 0.03 U/µl AccuStart Taq DNA polymerase (Quanta Biosciences), 0.01 U/µl T4 DNA ligase (Thermo Scientific) and 0.002 U/µl Uracil N-glycosylase (Thermo Scientific)). Quantitative PCR was performed in an MX3005 cycler (Stratagene) with an initial incubation at 95°C for 2 min, followed by 40 cycles of 95°C for 15 sec and 60°C for 1 min.

### Readout and data analysis of solution phase PLA

The results of solution phase PLA with real-time PCR readout were recorded using the MxPro software (Stratagene) and the cycle of threshold (Ct) was exported to Excel 2007 (Microsoft). The plots of data were either prepared in Excel 2007 (Microsoft) or using the statistical computing environment “R” (Bell Laboratories). The LOD was defined as the concentration of protein corresponding to a Ct value two standard deviations above the average assay background. The amplification template numbers N were calculated from Ct values using N = 2^40−Ct^, assuming that the Ct value for one molecule of template was 40. Coefficients of variation (CV) were calculated as CV% = StDev (N)/mean (N)×100%, where StDev and mean is the standard deviation and mean of quadruplicates of each sample.

### *In situ* PLA and immuno-RCA

Ten thousand SK-OV-3 cells were seeded per well on Lab-Tek Chamber Slides (154534, Thermo Scientific), and cultivated overnight. Cells were fixed with 3.7% paraformaldehyde for 30 min and permeabilized in PBS containing 0.1% Triton X-100 for 15 min. To block unspecific binding of the probes 1× blocking buffer (Olink Bioscience) was added to the cells and incubated for 1 h at 37°C. The blocking buffer was replaced by 40 µl of the purified and unpurified probes (20 nM of each purified 9.01-SNAP-S3primer_long and G3-SNAP-S3block_long conjugates for PLA; 5 nM of purified G3-SNAP-S3block_long conjugates and unpurified G3-SNAP-S3block conjugates for immuno rolling circle amplification (RCA)), diluted in the diluent buffer (Olink Bioscience). The probes solution was incubated with the cells for 1.5 h at 37°C, followed by two washes with washing buffer (DUO82049, Olink Bioscience). *In situ* PLA detection was then completed using the Duolink *in* *situ* detection kit Orange (excitation 554 nm/emission 576 nm, DUO92007, Olink Bioscience). For immuno-RCA the incubation with G3-SNAP conjugates was followed, after two washes, by a step where a 40 µl ligation mix (40 mM Tris-HCl, 10 mM MgCl_2_, 0.1 mM ATP, 100 nM S3 padlock oligonucleotide and 0.02 U/µl T4 DNA ligase (Thermo Scientific)) was added for a 30 min incubation at 37°C. After ligation and two washes, 40 µl amplification and detection mix (Duolink *in* *situ* detection kit Orange, DUO92007, Olink Bioscience) was applied to complete the immuno-RCA detection. Next, all slides were incubated with 1.25 mg/ml Hoechst 33342 (Life Technologies) for 20 min at RT to counterstain cell nuclei. The slides were then washed twice for 10 min in 1× buffer B (DUO82049, Olink Bioscience), followed by a 1 min wash step in 0.01× buffer B and mounting with Vectashield (H-1000, Vector Labs). In parallel, the cells on slides were analyzed with *in* *situ* PLA using 20 nM of pure G3-SNAP and 9.01-SNAP probes spiked with 20 nM of unconjugated G3-SNAP and 9.01-SNAP. The cells were also detected with immuno-RCA using 5 nM of G3-SNAP probe spiked with unconjugated G3-SNAP at different concentrations (40, 80, 160 or 320 nM).

### Imaging and data analysis

Cell images were acquired using an Axioplan 2 epifluorescence microscope (Zeiss), equipped with a 100 W mercury lamp, a cooled CCD camera (AxioCam HRm, Zeiss), a computer-controlled filter wheel with excitation and emission filters for visualization of DAPI (excitation 350 nm/emission 470 nm) and Cy3 (excitation 550 nm/emission 570 nm), and a 20× objective (Plan-Apochromat 440640-9903, Zeiss). Images were collected as compilations of Z-stacks and processed with AxioVision software (version 4.8, Zeiss). Image analysis and quantification of RCA products were conducted with the Duolink Image Tool (DUO90806, Olink Biosciences).

## Results and Discussion

**Figure 1:**
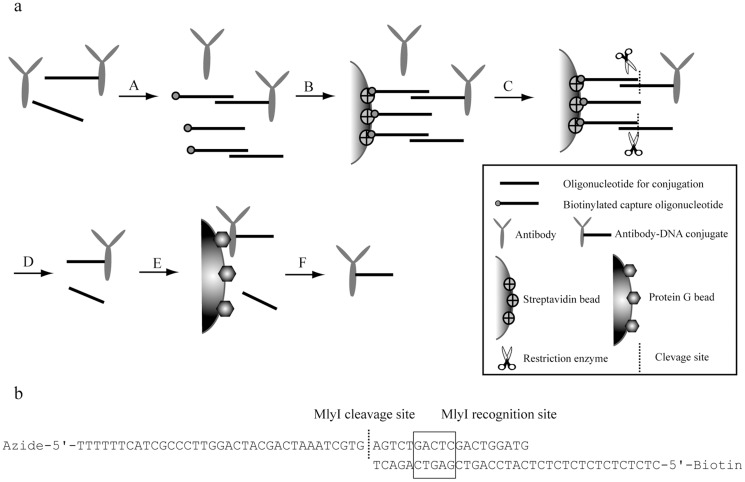
Schematic illustration of two-step purification of affinity binder-oligonucleotide conjugates. (a) Antibody-DNA conjugates illustrated here as an example. Conjugation of antibodies and oligonucleotides yields a mixture of desired conjugates, along with unconjugated antibodies and unconjugated oligonucleotides. (A) Biotinylated capture DNA oligonucleotides are hybridized to the oligonucleotides in the mixture. (B) Streptavidin-coated Sepharose beads are used to capture the biotinylated capture oligonucleotides, both in the form of conjugates and free oligonucleotides. (C) The unconjugated antibodies are removed by washes. (D) The MlyI enzyme is used to cleave the captured oligonucleotide hybrids, allowing both conjugates and oligonucleotides to be eluted from the solid support. (E) The eluate is then incubated either with protein G beads (for antibody-DNA conjugates) or with Dynabeads His tag (for DARPin-DNA conjugates), while free oligonucleotides are removed by washes. (F) Finally, purified conjugates are eluted from the solid support. (b) Illustration of hybridization of Arm1_long and Arm1 Capture, and the subsequent MlyI cleavage.

The general approach to prepare high-grade DNA-labeled affinity binders is schematically illustrated in Figure 1, exemplified with the preparation of antibody-oligonucleotide conjugates. First, the conjugation was performed in solution by mixing DBCO-modified antibodies with a 4-fold molar excess of azide-modified oligonucleotides. After incubation, the resulting mix containing antibody-DNA conjugates and remaining free antibodies and oligonucleotides, was incubated with biotinylated capture oligonucleotides at 2-fold molar excess over conjugation oligonucleotides, and the products were immobilized on streptavidin-coated Sepharose beads. After washes to remove unconjugated antibodies, the captured antibody-DNA conjugates along with unconjugated oligonucleotides were both released by enzymatic cleavage using the restriction enzyme MlyI that cleaves at the border of the complementary region between the capture and conjugate oligonucleotides (Fig. 1b) to release single-stranded DNA-coupled conjugates. The eluate was then captured on protein G beads and washes were conducted to remove any free oligonucleotides having no antibodies attached. Finally, purified conjugates were eluted from the protein G beads by lowering the pH.

In order to monitor the purification and MlyI cleavage steps, the antibodies were also conjugated separately to two shorter oligonucleotides (Arm1 and Arm2) corresponding precisely to the cleaved form. When these conjugates with shorter oligonucleotides were electrophoresed, they resulted in bands of identical molecular weight as the conjugates released by restriction digestion.

**Figure 2:**
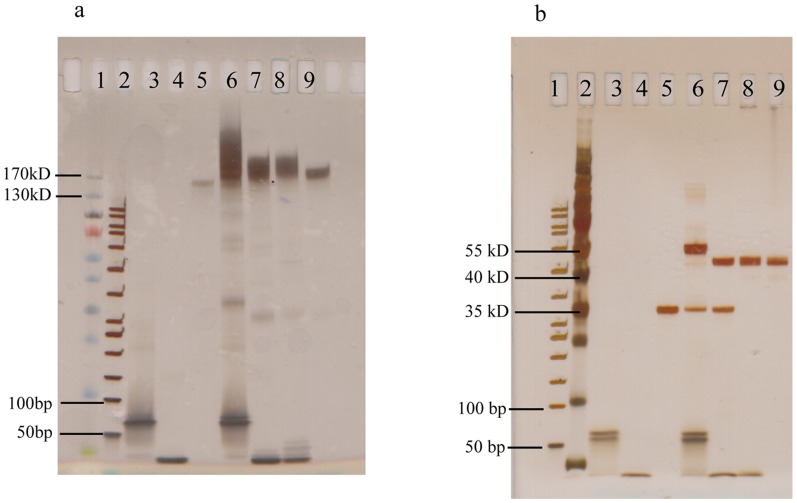
Gel electrophoresis of samples undergoing two-step purification. (a) Polyacrylamide gel of samples from purification of antibody-DNA conjugates and controls. Lane 1: PageRuler protein ladder (10–170 kDa, Thermo Scientific), Lane 2: 50 bp DNA ladder (Thermo Scientific), Lane 3: Arm1_long oligonucleotides (54 bp), Lane 4: Arm1 oligonucleotides (35 bp), Lane 5: anti-IL8 antibodies, Lane 6: conjugation reaction mixture of anti-IL8 and Arm1_long, Lane 7: conjugation reaction mixture of anti-IL8 and Arm1, Lane 8: eluate of captured sample of lane 6 after MlyI cleavage, Lane 9: anti-IL8 conjugates after two-step purification. The gel was stained with PlusOne DNA Silver Staining Kit. (b) Polyacrylamide gel of samples from purification of DARPin-DNA conjugates and controls. Lane 1: PageRuler protein ladder, Lane 2: 50 bp DNA ladder, Lane 3: S3block_long oligonucleotides (51 bp), Lane 4: S3block oligonucleotides (29 bp), Lane 5: G3-SNAP (34.5 kDa), Lane 6: conjugation reaction mixture of G3-SNAP and S3block_long, Lane 7: conjugation reaction mixture of G3-SNAP and S3block, Lane 8: eluate of sample of lane 6 after MlyI cleavage, Lane 9: G3-SNAP conjugates after the two-step purification. Relevant protein and DNA sizes are indicated. The gel was stained with Silver Stain for Mass Spectrometry kit.

The conjugation reaction mixtures, the eluates after enzymatic cleavage, and the purified conjugates were all analyzed by polyacrylamide gel electrophoresis and visualized with silver staining (Figure 2a). The conjugation reaction mixtures (lanes 6 and 7) resulted in a smear of bands similar to and larger than unconjugated antibodies (around 150 kDa, lane 5) and bands at the bottom of the gel corresponding to sizes of unconjugated DNA oligonucleotides (lane 3 and 4). After MlyI cleavage and elution, the cleaved excess oligonucleotides in the eluate from antibodies conjugated to Arm1_long (lane 8) migrated similarly to Arm1 (lane 4), which corresponds to the length of the cleaved products. This indicates that the MlyI cleavage works efficiently on the solid support. Under the native conditions that the samples were electrophoresed, the migration of conjugated antibodies in lanes 8 and 9 (around 170 kDa or larger) was only slightly slower than that of unconjugated antibodies (around 150 kDa, lane 5). After the whole purification protocol (Fig. 1) the oligonucleotides observed in the reaction mixture at the bottom of lane 8 were completely removed, and pure antibody-DNA conjugates were obtained and visualized (lane 9). The yield for antibody conjugates after the two-step purification was approximately 20% of the starting antibody input.

To investigate if a variation of the purification method would also be suitable for recombinant affinity reagents, a procedure similar to the one described above was performed for DARPin-SNAP constructs (HER2-specific G3-SNAP and 9.01-SNAP) [14], [15]. DARPins were expressed in fusion with a His-tag and a SNAP protein domain, allowing the convenient conjugation and purification of the DARPins. After reduction, the reactive cysteine residue within the SNAP domain readily reacts with benzylguanine (BG) to generate a covalent bond while releasing a guanine group [8]. The DARPin-SNAP constructs were first reduced with DTT and then reacted with BG-modified oligonucleotides to form DARPin-SNAP-DNA conjugates in solution [13]. To remove any remaining unconjugated protein, DARPin-SNAP-DNA conjugates were immobilized by hybridization to biotinylated oligonucleotides bound to streptavidin-coated Sepharose beads, in a manner similar to that used for the purification of antibody-DNA conjugates, and unconjugated DARPins were washed away. After restriction digestion with MlyI, the conjugated protein was bound to cobalt-chelating Dynabeads for his-tag isolation, and excess free oligonucleotides were then removed by washing. The pure DARPin-SNAP conjugates were finally eluted in a yield of around 60% out of the starting material.

To monitor the purification, the unpurified G3-SNAP conjugation reaction mixtures, eluates after enzymatic digestion, and the pure G3-SNAP conjugates were subjected to electrophoresis (as described above) and visualized with protein silver staining (Figure 2b). G3-SNAP conjugates with S3 block_long oligonucleotides (approximately 55 kDa, lane 6) and S3 block oligonucleotides (approximately 50 kDa, lane 7) showed intense bands at positions expected for the conjugates, in comparison to the unconjugated G3-SNAP (35 kDa, lane 5). Despite the use of a three-fold molar excess of oligonucleotides in the conjugation reaction, some remaining unconjugated G3-SNAP was nonetheless seen in the conjugation reaction mixture of G3-SNAP and S3 block_long (Fig. 2b, lane 6) and of G3-SNAP and S3 block (Fig. 2b, lane 7). The excess of oligonucleotides was also observed at the bottom of the gel (lane 6 and lane 7). After MlyI digestion, the eluate (lane 8) was visualized as two bands, corresponding to the conjugate and excess free S3 block oligonucleotides (which had been cleaved from excess S3 block_long). This demonstrated reliable cleavage by the MlyI restriction enzyme and efficient removal of unconjugated G3-SNAP. Finally, as a result of the two-step purification, a single, clean band of pure G3-SNAP-DNA conjugates was seen (lane 9), confirming that both free oligonucleotides and unconjugated G3-SNAP had been removed.

Homogenous – washing-independent – proximity ligation and extension reactions in solution have proven useful to analyze proteins in biofluids [9], [11]. Solution phase PLA and PEA require 1 µl of sample, and detection depends on dual recognition by oligonucleotide-modified affinity reagents, followed by DNA amplification via quantitative PCR. By suitable design of the DNA strands, it is possible to avoid detection signals from non-cognate pairs of antibodies or affinity reagents, thus avoiding increasing problems with cross-reactivity upon multiplexing. Accordingly, assays are available for up to 96 proteins in the same reaction, with performance similar to that of individual assays [16], [17]. Since the homogeneous proximity reactions (PLA and PEA) do not involve any washing steps, it is necessary to use high-quality probes where both unconjugated antibodies and free oligonucleotides have been removed, as they would otherwise be expected to compromise the performance of solution phase reactions.

**Figure 3:**
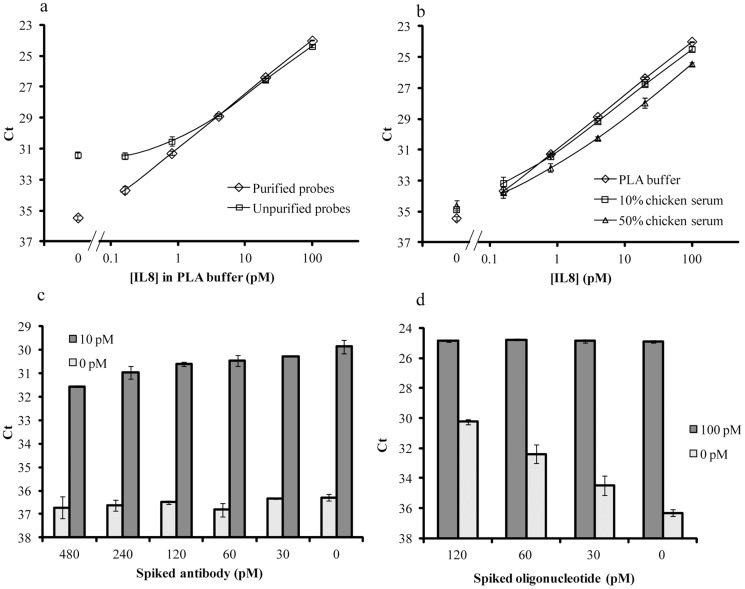
Solution phase PLA for detection of recombinant human IL8. (a) Detection of IL8 in PLA buffer with 60 pM pure anti-IL8 DNA conjugates (diamonds) and 60 pM unpurified anti-IL8 DNA conjugates (squares). (b) Detection of IL8 in PLA buffer (diamonds), 10% chicken serum (squares) and 50% chicken serum (triangles) using 60 pM pure anti-IL8 DNA conjugates. (c) Different amounts of unconjugated anti-IL8 (at a final concentration from 480 pM to 0 pM) were spiked in 60 pM of pure anti-IL8 conjugates. This series of mixes of probes were used in samples with either 10 pM or 0 pM IL8 in PLA buffer. (d) Different amounts of unconjugated oligonucleotides (Arm1 and Arm2 at a final concentration from 120 pM to 0 pM) were spiked in 60 pM of pure anti-IL8 conjugates. The probe mixes were employed in samples with 100 pM or 0 pM IL8 in PLA buffer. Error bars (a and b) indicate standard deviation from quadruplicate reactions and error bars (c and d) indicate standard deviation from duplicate reactions.

To assess the effect of purifying the conjugates, we compared the performance of pure and unpurified conjugates (probes) in assays for detection of human interleukin 8 (IL8) protein using solution phase PLA. The reactions were performed in PLA buffer alone or with 10% or 50% chicken serum. The purified conjugates resulted in a limit of detection (LOD) of 0.06 pM, which represents an approximately 16-fold increased sensitivity of detection over the unpurified conjugates (0.97 pM) (Figure 3a, Table 1). In complex samples with 10% or 50% chicken serum, solution phase PLA probed with pure conjugates demonstrated robust and reproducible measurement of IL8 protein (Figure 3b, Table 1), and the chicken serum did not impair the LOD of the assay or its linear range compared to the corresponding assay in PLA buffer (Figure 3b).

**Table 1:** Summary of solution phase PLA for detection of IL8 proteins in PLA buffer or in 10% or 50% chicken serum.

Probes	Matrices	R^2^	LOD (pM)	Background Ct	Background CV%
Unpurified	Buffer	0.9773	0.97	31.39	14.1%
Purified	Buffer	0.9999	0.06	35.43	9.0%
Purified	10% Chicken serum	0.9969	0.26	34.87	13.7%
Purified	50% Chicken serum	0.9921	0.17	34.62	19.5%

R^2^ is the correlation coefficient of the fitting curve from 100 pM to 0.16 pM. Background Ct equals the average Ct from quadruplicates of sample in reactions with no spiked IL8. Background CV% is the coefficient of variation calculated from the background Ct values for all quadruplicates.

Next, we sought to determine to what extent unconjugated antibodies or free oligonucleotides would interfere with the performance of PLA in solution. We assayed samples containing 10 pM IL8 in PLA buffer using purified conjugates to which different amounts of unconjugated antibodies specific for IL8 or unconjugated oligonucleotides (Arm1 and Arm2) had been added. Unconjugated antibodies at a concentration of 480 pM in assays using 60 pM conjugates caused a 2-fold decrease in detection signals (Figure 3c). This is in accordance with the expectation that unconjugated antibodies would compete with the PLA probes for binding to their cognate targets and thus reduce detection efficiency. Free oligonucleotides spiked at a final concentration of 30 pM into detection reactions with 100 pM IL8 increased assay background by approximately two Ct units (Figure 3d), indicating that free oligonucleotides in the reactions cause an increased assay background and lead to a decreased signal-to-noise ratio by around 4-fold at these concentrations.

**Figure 4:**
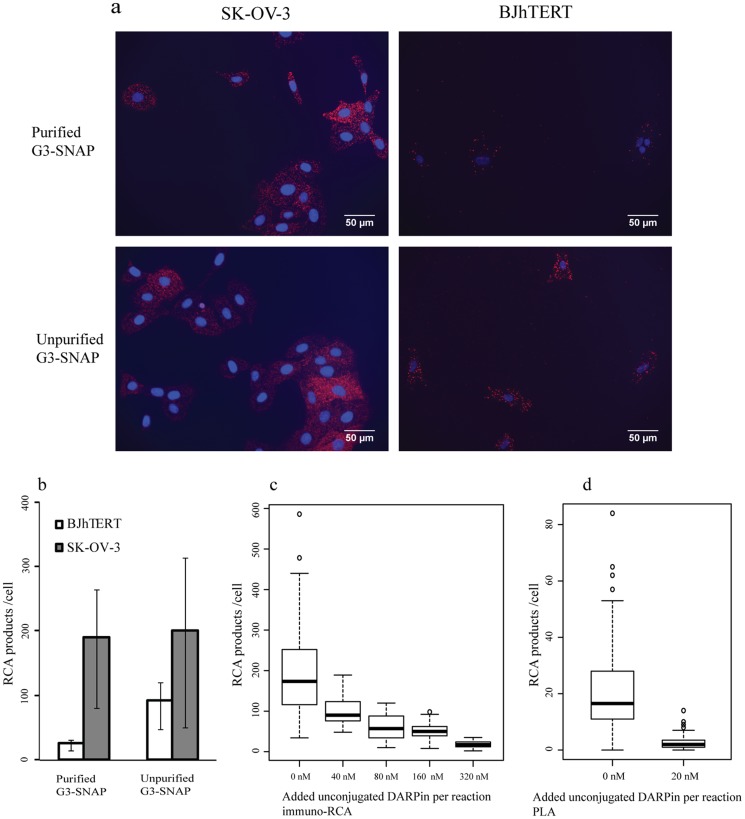
Detection of HER2 proteins using immuno-RCA and *in* *situ* PLA in fixed cells. (a) 5 nM pure G3-SNAP conjugates and 5 nM unpurified G3-SNAP conjugates were separately used in immune-RCA to detect HER2 proteins in HER2-positive (SK-OV-3) or -negative (BJhTERT) cells. Red dots represent RCA products arising from immuno-RCA reactions detected with fluorescence labeled complementary oligonucleotides. The numbers of RCA products per cell from those images are shown in bar charts (b). (c) Boxplots present the numbers of RCA products per cell when mixes of pure G3-SNAP conjugates (5 nM) and different amounts of unconjugated G3-SNAP (from 0 nM to 320 nM) were used to detect HER2 proteins via immuno-RCA in SK-OV-3 cells. (d) Boxplots reflect the number of RCA products per cell after *in* *situ* PLA for detection of HER2 protein in SK-OV-3 cells, probed with pure probe conjugates (20 nM G3-SNAP conjugates and 20 nM 9.01-SNAP conjugates), mixed with different amounts of unconjugated DARPin binders (G3-SNAP and 9.03-SNAP at concentrations of 0 nM or 20 nM). The cell nuclei were counterstained with DAPI (blue).

We also investigated the effects of impure probes on *in* *situ* protein detection reactions. The purified DARPin-DNA conjugates were used alongside an unpurified DARPin-DNA conjugate in immuno-RCA to detect HER2 proteins in fixed SK-OV-3 cells abundantly expressing HER2 protein [18] and BJhTERT cells lacking the protein [19]. At a probe concentration of 5 nM the pure G3-SNAP conjugates resulted in a 3-fold lower background than the unpurified G3-SNAP conjugates in BJhTERT cells (Figure 4a and b). The increased background from unpurified probes in BJhTERT cells probably resulted from any free oligonucleotides that may resist washes. The signal-to-noise ratio, the number of RCA products in SK-OV-3 cells divided by those in BJhTERT cells, was lower in assays using unpurified probes compared to those using purified conjugates (Figure 4a and b). This is likely a consequence both of unconjugated affinity reagents (G3-SNAP) blocking target molecules and free oligonucleotides resistant to washes that may contribute to nonspecific signals. In order to assess the effect of free affinity reagents in these assays, G3-SNAP was added back to the purified G3-SNAP conjugates that were used both in immuno-RCA and *in* *situ* PLA. As shown in Figure 4c, the presence of 40 nM free G3-SNAP greatly reduced detection signals of HER2 proteins in SK-OV-3 cells. At a concentration 64-fold higher than that of the conjugates, unconjugated DARPins abolished detection of HER2 proteins in SK-OV-3 cells (Figure 4c). The numbers of detection signals for HER2 proteins using *in* *situ* PLA decreased by a factor of four when the purified conjugates of either G3-SNAP or 9.01-SNAP were mixed with an equal concentration of the corresponding free affinity reagents (Figure 4d), underlining the importance of purified detection reagents.

In summary, we have demonstrated a generalized and robust approach for preparing high quality oligonucleotide-modified affinity reagents, using either antibodies or recombinant binders. The obtained oligonucleotide-conjugated affinity reagents exhibited greatly improved performance in protein analysis using DNA-assisted protein detection reactions. The two-step purification on solid supports is suitable for scalable, high-throughput preparation of high-grade oligonucleotide-conjugated antibody-based or recombinant affinity reagents.

## Supporting Information

Table S1 **Oligonucleotide sequence and modification.**
(DOCX)
