# Supplementary material for: A Universal Approach to Prepare Reagents for DNA-Assisted Protein Analysis
Source: PLoS One. 2014 Sep 18;9(9):e108061. doi: 10.1371/journal.pone.0108061 (PMC4169473; doi:10.1371/journal.pone.0108061)
Supplement: Table S1 — Oligonucleotide sequence and modification. (DOCX) [file pone.0108061.s001.docx]

**Table S1 Oligonucleotide sequence and modification**

| **Name** | **5' Modification** | **3' Modification** | **Sequence 5'-->3'** |
| --- | --- | --- | --- |
| Arm1_long | Azide | None | ttttttCATCGCCCTTGGACTACGACTAAATCGTGAGTCTGACTCGACTGGATG |
| Arm2_long | None | Azide | GACTGGATGGAGTCAGACTTCGTGTCTAAAGTCCGTTACCTTGATTtctctc |
| Arm1 | Azide | None | ttttttCATCGCCCTTGGACTACGACTAAATCGTG |
| Arm2 | Phosphate | Azide | TCGTGTCTAAAGTCCGTTACCTTGATTtctctc |
| Arm1Capture | Biotin | None | CTCTCTCTCTCTCTCTCATCCAGTCGAGTCAGACT |
| Arm2Capture | None | Biotin | AGTCTGACTCCATCCAGTCTCTCTCTCTCTCTCTC |
| S3primer_long | Amino Modifier C6 | None | AAAAAAAAAACATATGACAGAACTAGACACTCTTAGTCTGACTCGACTGGATG |
| S3block_long | Amino Modifier C6 | None | AAAAAAAAAAGACGCTAATAGTTAAGACGCTTAGTCTGACTCGACTGGATG |
| S3primer | Amino Modifier C6 | None | AAAAAAAAAACATATGACAGAACTAGACACT |
| S3block | Amino Modifier C6 | None | AAAAAAAAAAGACGCTAATAGTTAAGACG |
| S3Capture | Biotin | None | CTCTCTCTCTCTCTCTCATCCAGTCGAGTCAGACTAA |
| BioFwd | None | None | CATCGCCCTTGGACTACGA |
| BioRev | None | None | GGGAATCAAGGTAACGGACTTTAG |
| BioSplint | None | None | TACTTAGACACGACACGATTTAGTTT |
| S3 padlock | Phophate | None | CTATTAGCGTCCAGTGAATGCGAGTCCGTCTAAGAGAGTAGTACAGCAGCCGTCAAGCGTCTTAA |
